# Novel Insights into RAD52’s Structure, Function, and Druggability for Synthetic Lethality and Innovative Anticancer Therapies

**DOI:** 10.3390/cancers15061817

**Published:** 2023-03-17

**Authors:** Beatrice Balboni, Francesco Rinaldi, Viola Previtali, Andrea Ciamarone, Stefania Girotto, Andrea Cavalli

**Affiliations:** 1 Computational and Chemical Biology, Istituto Italiano di Tecnologia, via Morego 30, 16163 Genoa, Italy; 2 Department of Pharmacy and Biotechnology, University of Bologna, via Belmeloro 6, 40126 Bologna, Italy; 3 Structural Biophysics and Translational Pharmacology Facility, Istituto Italiano di Tecnologia, via Morego 30, 16163 Genoa, Italy

**Keywords:** RAD52, synthetic lethality, precision medicine, drug discovery

## Abstract

**Simple Summary:**

Human RAD52 is a non-essential DNA/RNA-binding protein thought to be involved in many DNA repair mechanisms. Initially regarded as having a major role only in error-prone backup DNA repair mechanisms, RAD52 has recently gained attention because its inhibition induces synthetic lethality in cancer cells with an inactivated homologous recombination pathway (for error-free double-strand-break repair). RAD52 is thus a potential target to overcome resistance and unwanted side effects. Unfortunately, researchers still lack detailed structural and mechanistic information on RAD52 and have identified only a limited number of inhibitors, none of which are in the preclinical phase. This review summarizes the current knowledge on RAD52, highlighting the potential of its inhibition. This review also discusses the critical gaps in knowledge and sets out future directions for effective campaigns to discover RAD52 inhibitors.

**Abstract:**

In recent years, the RAD52 protein has been highlighted as a mediator of many DNA repair mechanisms. While RAD52 was initially considered to be a non-essential auxiliary factor, its inhibition has more recently been demonstrated to be synthetically lethal in cancer cells bearing mutations and inactivation of specific intracellular pathways, such as homologous recombination. RAD52 is now recognized as a novel and critical pharmacological target. In this review, we comprehensively describe the available structural and functional information on RAD52. The review highlights the pathways in which RAD52 is involved and the approaches to RAD52 inhibition. We discuss the multifaceted role of this protein, which has a complex, dynamic, and functional 3D superstructural arrangement. This complexity reinforces the need to further investigate and characterize RAD52 to solve a challenging mechanistic puzzle and pave the way for a robust drug discovery campaign.

## 1. Introduction

RAD52 is a DNA/RNA-binding protein that plays a multifaceted role in many intracellular pathways related to DNA repair and the maintenance of genomic stability [1,2]. Until recently, RAD52 was not thought to be a potential pharmacological target but rather an auxiliary protein that was non-essential in living cells [3,4]. However, RAD52 has since been reevaluated as a potential target for cancer treatment. Indeed, until 2019, it was previously of interest for its single-strand annealing (SSA) activity [1,2,5]. Since then, RAD52 has been proposed as a novel and attractive pharmacological target to boost chemical-induced synthetic lethality therapies, avoiding resistance and severe side effects [1,2]. However, screening campaigns to discover RAD52 inhibitors are challenging because of the earlier lack of interest in this multifaceted and complex target. Indeed, while RAD52’s involvement in multiple pathways is fascinating, detailed knowledge of these roles is required to avoid unwanted side effects. Moreover, RAD52’s dynamic 3D superstructure is difficult to investigate, handle, and target. Therefore, even though all the evidence suggests that RAD52 is a promising pharmacological target for cancer therapies, researchers still have very limited knowledge about this multifaceted protein. More knowledge is needed to set up an effective drug discovery campaign. In recent years, great effort has been made to fill the RAD52 knowledge gap and better describe its features and mechanistic details in order to design promising strategies for RAD52 inhibition.

This review will describe the current knowledge of RAD52’s main structural and functional hallmarks and report its current involvement in cancer therapies. Moreover, we will describe the few available RAD52 inhibitors and discuss RAD52’s role as a promising novel pharmacological target in synthetic lethality strategies. By comprehensively describing the available information on RAD52, we will highlight the critical aspects of this target and the knowledge gaps that must be filled to enable more robust drug discovery campaigns.

## 2. RAD52 Functions

### 2.1. Homologous Recombination (HR)

RAD52 is a key mediator of the HR-based DNA repair mechanism in yeast. RAD52 has a key role in recruiting RAD51 on Double Strand Break (DSB) sites and in promoting the annealing of ssDNA complexed with Replication Protein A (RPA), thus facilitating RAD51 recombinase activity [6,7,8]. In vertebrates, however, RAD52 appears to be only an auxiliary redundant factor in the RAD51-dependent HR pathway, where its inactivation does not induce any significant cellular impairment. In vivo experiments have suggested that RAD52-null mice are viable with no evident phenotype [3], while experiments on DT40 chicken B cells (DT40) showed that inactivating RAD52 does not affect viability or cell health, with a RAD52-depleted phenotype comparable to a wild-type phenotype [4]. Specifically, RAD52 depletion in DT40 cells leads to only a slight reduction in targeted chromosomal integration without a detectable increase in radiation sensitivity, in contrast to what would be expected if RAD52 had an essential role in the HR mechanism, as it does in yeasts [4]. The apparent dispensability of RAD52 in vertebrates means that RAD52 was long overlooked as a pharmacological target.

The Powell group was the first to draw attention to human RAD52 when they showed that breast cancer type 2 susceptibility protein (BRCA2)-deficient cells require RAD52 for survival [9,10]. Indeed, they highlighted that RAD52 may have an important role as a backup for HR-related mediators in pathological conditions. Namely, RAD52 depletion in BRCA-deficient cells results in severe phenotype effects such as RAD51-impaired foci formation and genomic instability [9,10]. These data led to the hypothesis that RAD52 may be able to conduct RAD51 loading, albeit at lower efficiency, serving as a backup for BRCA2 in mediating RAD51 activity in HR [9,10]. This HR-related backup role in BRCA-deficient cells was also supported by a recent study by Mahajan and colleagues [11]. In agreement with previous observations, they found that RAD52 overexpression rescues the excessive origin firing and checkpoint control defects observed in BRCA2-deficient cells, compensating for BRCA2 loss. Moreover, RAD52 interacts with phosphorylated Checkpoint Kinase 1 (pCHK1), acting as BRCA2 for HR regulation and maintaining checkpoint control during DNA damage response [11]. However, all these hypotheses need to be further supported by in vitro and in vivo studies to be validated.

Through an in vitro study using a system that mimics the key steps in the recombination process, it has also been suggested that RAD52 may have a role in the HR mechanism’s second-end capture [12]. This step takes place right after the D-loop formation at the DSB site, upon completion of the DNA synthesis. The DSB’s second end, leading to the formation of Holliday Junctions (HJs) intermediates in the HR process, would be captured via RAD52 annealing to the D-loop. Rad52′s potential involvement in the HR second-end capture has also been later reported in yeast, but, again, through in vitro assays [13]. Even though the ssDNA binding and annealing activities of RAD52, in the presence of RPA, prove its ability to play a postsynaptic role in HR by annealing the second end of a DSB to an extended D loop, no in vivo proof is available yet.

Despite RAD52’s clear involvement in the HR pathway, researchers lack the information necessary to comprehensively describe its role.

### 2.2. Single-Strand Annealing (SSA)

RAD52’s most studied function is as the key mediator of the SSA DNA repair mechanism. SSA is an error-prone mechanism used when DSBs occur in highly repeated DNA regions [14,15]. SSA relies on long homology tracts to anneal two 3′-ssDNA overhangs together. It thus requires neither a template donor from a sister chromatid nor strand invasion, in contrast to RAD51-dependent HR [1].

**Figure 1:**
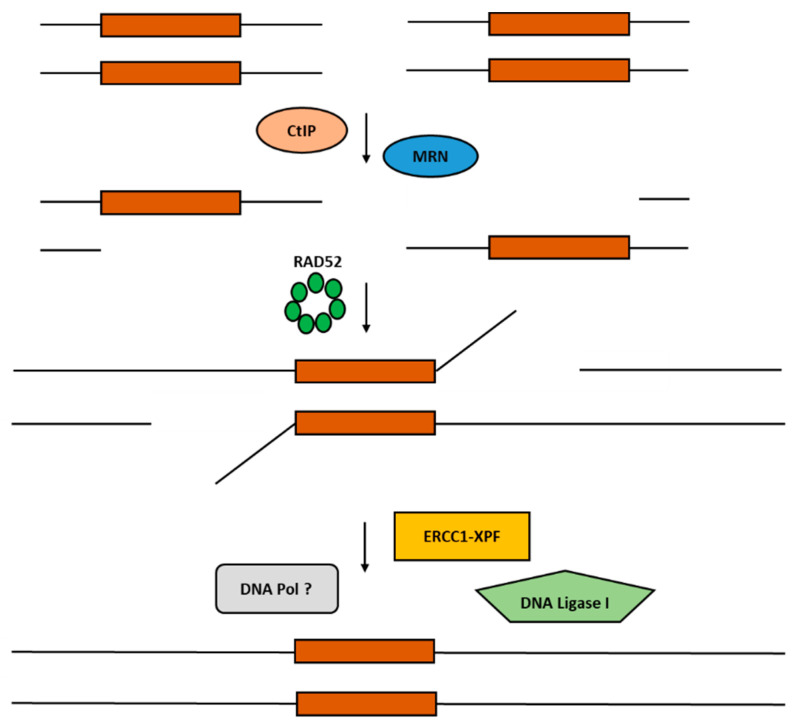
Schematic representation of the RAD52-mediated SSA DNA repair mechanism. RAD52 facilitates homology search and strand annealing.

As shown in Figure 1 and such as HR, SSA begins with the resection of DNA ends and the creation of 3′ overhangs thanks to the nuclease activity of Mre11/Rad50/Nbs1 (MRN) and C-terminal binding protein 1 (CtBP1) interacting protein (CtIP) complexes [15,16,17]. Additionally, other helicases and nucleases can be recruited to generate longer single stretches (e.g., DNA2, Bloom Syndrome Protein (BLM), Exonuclease I (EXO1), Werner Syndrome Helicase (WRN), and RecQ-Like Helicase) [18]. RAD52 is then recruited on the resected DNA ends to promote their annealing and to recognize the ssDNA region of homology (<30 bps). Following the annealing in the homology regions, the Excision Repair Cross-Complementation Group 1 (ERCC1)-Xenoderma Pigmentosum Complementation Group F (XPF) complex binds RAD52’s N-terminal domain and cleaves nonhomologous 3′-ssDNA flap ends [19,20]. RAD52 seems to stimulate ERCC1-XPF nuclease activity [19]. Then, DNA polymerases fill any gaps, and DNA ligase I blends the DNA strands to complete the SSA process. Researchers have not yet identified and characterized all the polymerases and ligases involved in SSA [14]. 

Interestingly, novel insights have demonstrated that RAD52’s SSA activity in vitro may be enhanced by its direct binding to the very acidic protein Deleted in Split Hand/Split Foot 1 (DSS1) [21]. This interaction is believed to change RAD52’s conformation and modulate its binding to DNA, inducing a four-fold increase in SSA efficacy, mainly due to the higher annealing rate of RAD52-ssDNA. Actually, from the in vitro model, the DSS1-RAD52 interaction would allow rescue for BRCA2-deficiency in cancer cells by promoting SSA and break-induced replication (BIR) (see below) activities mediated by RAD52. Nevertheless, these potential RAD52 activities have not yet been confirmed in vivo.

### 2.3. Stalled Replication Fork

During DNA replication, cells can incur DNA damage. This damage can take place at the replication fork site, leading to a stall and, if prolonged, a fork collapse. Cells have a number of mechanisms to recover from DNA lesions that stall DNA replication forks [22,23]. These recovery mechanisms fall into the categories of damage bypass, fork reversal, and fork breakage [23]. RAD52 seems to have important roles in solving these types of stress-replication structures both upstream and downstream of the fork replication remodeling [23].

**Figure 2:**
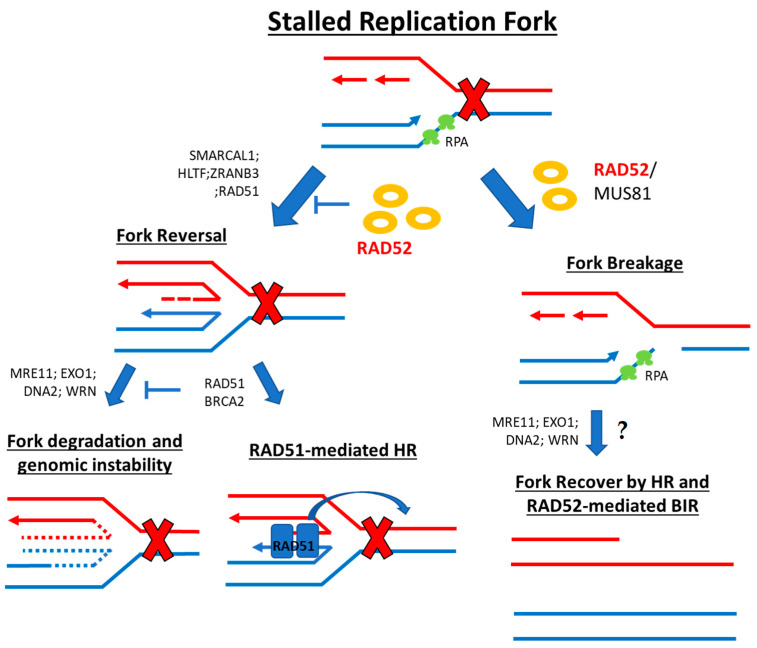
Schematic representation of stalled replication fork steps involving RAD52. (**Left**): RAD52 acts as a gatekeeper for the replicative fork to prevent unscheduled MRE11-mediated degradation and to facilitate enzyme loading only when required. (**Right**): RAD52 mediates the fork break mechanism for stall resolution and may mediate fork recovery through BIR.

Fork reversal events allow the cell to bypass DNA damage by incorporating the correct nucleotides and using the newly synthesized sister strand as a template instead of the lesion-containing strand (i.e., “chicken foot” formation) (Figure 2). This process involves the highly regulated interplay and counterbalancing of the effects of many players, such as ssDNA-binding proteins and recombinases (RPA, RAD52, BRCA2, RAD51, RADX), translocases (SWI/SNF-related matrix-associated actin-dependent regulator of chromatin subfamily A-like protein 1 (SMARCAL1), Zinc Finger, RAN-Binding Domain Containing 3 (ZRANB3), Helicase-Like Transcription Factor (HLTF), SNF2 Histone Linker PHD RING Helicase (SHPRH), WRN, RECQ1, ATPase family AAA domain-containing protein 5 (ATAD5)), and exo-/endonucleases (Meiotic Recombination (MRE11), EXO1, DNA2, MUS81) [1]. RAD52 here is involved in fork protection upstream of the actual reversal mechanism, acting as a gatekeeper for the replicative fork state. This prevents unscheduled MRE11-mediated degradation and ensures that fork reversal enzymes load only when required [24] (Figure 2). RAD52 depletion or inhibition results in excessive loading of RAD51, SMARCAL1, and ZRANB3 at stalled replication forks, leading to unscheduled fork reversal and MRE11-dependent degradation that causes genome instability. Intriguingly, RAD52 also recruits MRE11-MUS81 to unprotected reversed forks in BRCA2-deficient pathological conditions [24,25]. Moreover, in CHK1-deficient cells where the G2/M cell cycle checkpoint is lost, cell survival is dependent on RAD52 and MUS81 to tackle replication stress [26]. In addition to these mechanisms, RAD52 also takes part in the fork breakage mechanism for fork stall resolution. Here, one of the fork arms is detached, leaving a one-ended DSB (Figure 2). These structures are then recovered by HR- and RAD52-mediated break-induced replication (BIR) and/or mitotic DNA synthesis (MiDAS) [1,23].

BIR: At collapsed replication forks, RAD52 can activate BIR. BIR is a specialized pathway that repairs single-ended DSBs and is well-characterized in yeast systems [27]. Briefly, after fork cleavage, at one end of the DSB, the end is resected, and RAD52 protein facilitates RAD51 filament formation on ssDNA. This nucleoprotein formation then invades the homologous region of the interacting sister strand, forming a D-loop. The replisome then assembles with Pol32 (DNA Polymerase Delta Subunit 3/DNA Polymerase Delta Subunit 4 (POLD3/POLD4) in humans [28,29]) and the DNA synthesis begins. Studies in mammalian cells report that RAD52 is necessary to facilitate DNA strand invasion to form a D-loop and to anneal DNA strands after recruitment of MUS81 and the endonuclease/exonuclease/phosphatase family domain containing 1 (EEPD1) nucleases to the collapsed fork [1,30].

MiDAS: MiDAS is a microhomology-mediated BIR that usually takes place in the presence of common fragile sites (CFS) [31] and other difficult-to-replicate regions in order to complete DNA replication before cell division. These are usually AT-rich long coding regions, in which transcribing RNA polymerases often collide with replicating DNA polymerase [32]. At colliding polymerases, the fork stalls, and the Fanconi Anemia Group D2 protein 2 (FANCD2)/Fanconi Anemia Group D2 protein 1 (FANC1) complex binds and tethers the sister chromatids. Here, RAD52 is thought to help microhomology-mediated annealing of DNA strands. The intermediate DNA structure is then processed by MUS81-EME1-SLX4 and other nucleases, and the DNA synthesis is performed by POLD3, as in BIR [31]. Moreover, RAD52 is thought to help the recruitment of MUS81 and POLD3 to CSFs in early mitosis [31]. Interestingly, if MiDAS fails to repair the DNA damage before cell division, the daughter cells inherit damaged DNA that is sequestered by 53BP1 bodies during the G1 phase [33]. In the following S phase, these formations can be dissolved by RIF1 activity, triggering RAD52 recruitment and leading to a second DNA repair mechanism through a BIR-equivalent pathway [34]. 

As already mentioned, RAD52’s SSA and BIR activity seems to be promoted by its interaction with the DSS1 protein, even though in vivo evidence is still lacking to corroborate the in vitro formulated hypothesis [21].

BIR-associated RAD52 activity was also recently reported in the alternative lengthening of telomeres (ALT). Zhang and colleagues demonstrated that RAD52 can directly promote D-loop formation in vitro and maintain telomere length in ALT-associated PML bodies (APBs) in ALT-proficient cells [35].

### 2.4. RNA-Dependent DNA Repair

Even though the HR mechanism for DNA repair is mostly active in the G2/S phase, an HR sub-pathway that uses RNA transcripts as an alternative template for DSB repair is active during the G0 and G1 phases. Notably, this mechanism can be independent of reverse transcriptases [2,5] and does not require a sister chromatid for the DNA template sequence. As such, RNA-dependent DNA repair also occurs in nondividing cells such as terminally differentiated neurons [36]. This mechanism has been described as RAD52-dependent in both yeast and human cells [37,38]. Specifically, RAD52 is thought to have two modes of action in this pathway. First, RAD52 allows homologous RNA transcripts to “bridge” DSB ends, leading to the formation of an RNA:DNA heteroduplex complex upon DSB at highly transcribed loci [38,39]. Here, RAD52 uses RNA to tether both ends of a homologous DSB, forming a DNA synapse for ligation and damage resolution. At the end of this process, RNA degradation by RNase H may also occur [40]. In 2017, Mazina and colleagues also studied this mode of action of RAD52 in RNA-dependent DNA repair. They showed that RAD52 allows strand exchange between ssRNA and dsDNA through an unconventional “inverse strand exchange”, forming a nucleoprotein complex with dsDNA and promoting strand exchange with ssDNA or ssRNA [39].

Secondly, an RNA-mediated DNA repair mechanism is thought to require RAD52 to promote the annealing between complementary ssDNA and template RNA [1,40]. In this model, RAD52 forms an RNA-DNA hybrid along the 3′ overhang of a DSB. The RNA is used as a template for DNA repair synthesis by reverse transcriptase (RT). The RNA is then degraded by RNase H, and RAD52 can promote SSA of the opposing homologous ssDNA overhangs. The final processing of the DSB involves gap filling and ligation [40].

In both mechanisms, it is thought that the RNA-dependent DNA synthesis for gap filling can be performed by specific DNA polymerases with specific reverse transcriptase activity (i.e., Polη and Polθ [41,42]).

RNA-dependent DNA synthesis in eukaryotes requires RAD52 and no other paralogs such as RAD59 or the recombinase activity of RAD51 [39,43]. 

The formation and resolution of R-loops (three-stranded DNA:RNA hybrids) are commonly involved in specific intracellular signal transduction for efficient DSB repair. Moreover, transcriptionally active genes preferentially recruit HR mediators compared to untranscribed genes [44]. This makes R-loop formation an important factor for the downstream recruitment of repair proteins. Notably, Tseng and colleagues suggested that RAD52 has a role in the RNA-dependent DNA repair mechanism called transcription-coupled homologous recombination (TC-HR) [45]. Here, RAD52 may be recruited at the R-loop by the Cockayne syndrome B (CSB) protein to help the loading of RAD51 onto the DSB site in a BRCA1-/BRCA2-independent manner [45].

Yasuhara and colleagues suggested a similar pathway, namely transcription-associated homologous recombination repair (TA-HRR) [46]. Here, DNA repair in highly transcribed regions requires R-loops and RAD52. RAD52 recruits XPG endonuclease, which processes R-loops into substrates with ssDNA overhangs, such as resected ends that can undergo HR mechanisms and DNA repair [46]. In contrast to TC-HR, TA-HRR involves BRCA1 and occurs in the S/G2 phase.

Interestingly, although RAD52’s preferential substrate remains ssDNA, it can directly bind to the R-loop or other DNA:RNA hybrids [36,39]. It is also worth mentioning that recent in vitro studies have shown that RAD52 has a better affinity for DNA:RNA hybrids containing m5C-modified RNA. It has been suggested that m5C mRNA modifications may take place at DNA-damaged sites to regulate DNA repair, thus further supporting RAD52 involvement in DNA repair [47].

Additional studies are required to further elucidate RAD52’s role in these RNA-dependent DNA repair mechanisms.

### 2.5. Regulatory Role

Wang and colleagues recently speculated on RAD52’s role in regulating the balance between mechanisms of single-strand-break repair (SSBR) and double-strand-break repair (DSBR) [48]. Specifically, in their in vitro studies, RAD52 inhibits SSBR through strong ssDNA and/or Poly-ADP Ribose Polymerase (PARP1) binding affinity, reducing DNA-damage-promoted X-ray Repair Cross Complementing 1 (XRCC1)/DNA Ligase £a (LIG3a) co-localization. RAD52’s inhibitory effects on SSBR neutralize RAD52’s role in DSBR in specific cellular damage conditions, suggesting that RAD52 may maintain a balance between cell survival and genomic integrity. Moreover, they also reported that the disruption of RAD52 oligomerization affects RAD52’s DSBR activity but not its ssDNA-binding ability, which is required for RAD52’s inhibitory effects on SSBR. Wang and colleagues, therefore, suggest a novel RAD52-inhibition-based strategy to sensitize cells to different DNA-damaging agents [48]. Nevertheless, additional pieces of evidence in cells are required to corroborate this hypothesis.

## 3. RAD52’s Structure

As discussed above, RAD52 is thought to be involved in many activities. This suggests that the protein has a dynamic nature, which is reflected in its complex and mobile 3D structure, as described below.

**Figure 3:**
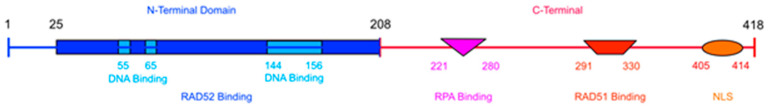
Domain map of human RAD52: the N-terminal domain contains the DNA-binding region and a self-associating region; the C-terminal domain contains RPA and RAD51 interacting regions and a nuclear localization signal.

**Figure 4:**
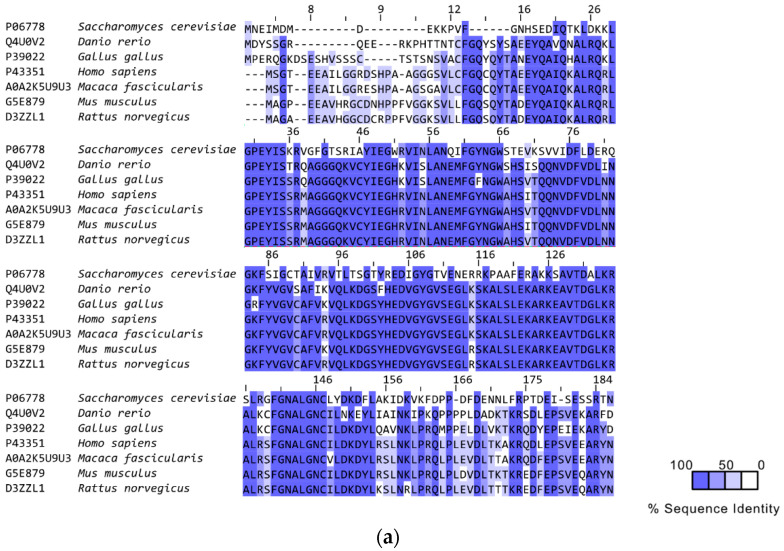

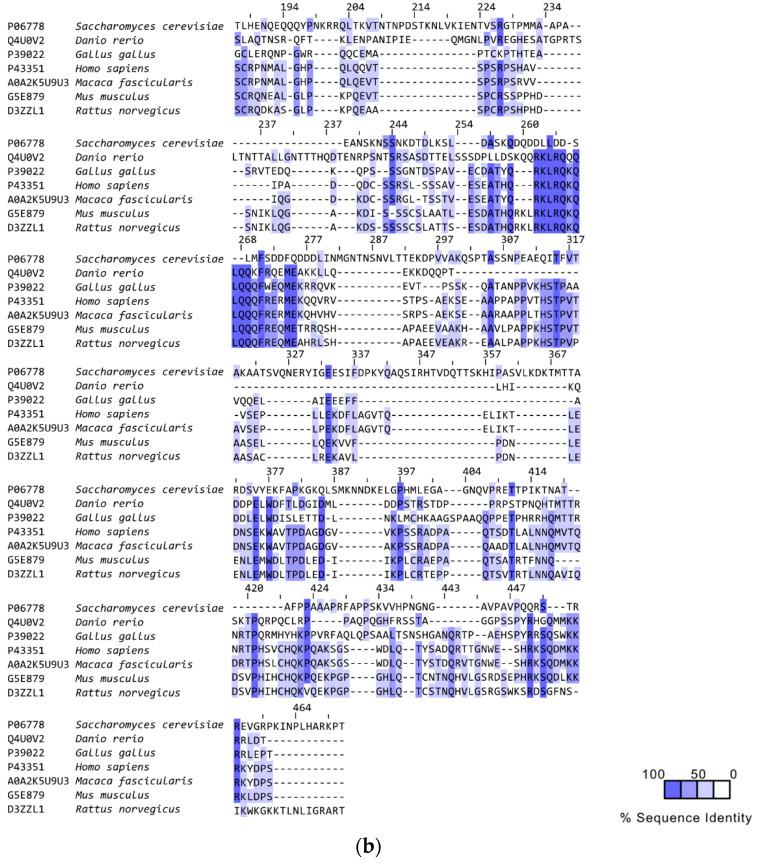
Multiple alignments of RAD52 sequences. (**a**) Alignment of N-terminal and (**b**) C-terminal domain sequences of different organisms. In contrast to the N-terminal domain, the C-terminal domain is not conserved.

Human RAD52 is a 47 kDa protein of 418 amino acids that form multimeric, ring-shaped functional units. Its N-terminal domain (1–208 AA) comprises the oligomerization domain and the DNA-binding domain and shares high homology with yeast Rad52 (>70% sequence homology [49,50]), whereas its C-terminal domain contains the RAD51 binding site and the Replication Protein A (RPA)-binding site and has only modest homology with yeast Rad52 (Figure 3 and Figure 4) [49,51].

Low-resolution evidence from electron microscopy studies on full-length RAD52 (RA52 FL) [52,53,54] suggests that RAD52 forms heptameric rings. Noteworthy, the same group recently published a cryogenic electron microscopy (cryo-EM) structure of RAD52 FL, showing that the full-length protein can oligomerize as an undecamer, suggesting a new hypothesis for the oligomerization state of RAD52 FL, in contrast to what was previously speculated [55].

**Figure 5:**
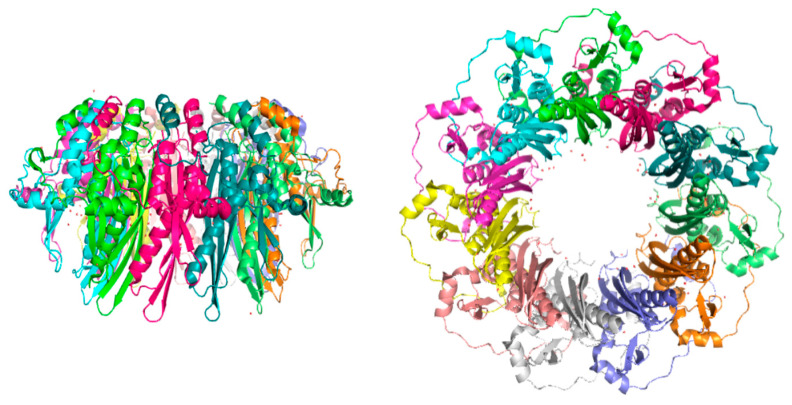
Side and bottom views of the mushroom-like structure of the undecameric ring of RAD52 (1–212) (PDB 1KN0). Molecular graphics and analyses were performed with UCSF Chimera, developed by the Resource for Biocomputing, Visualization, and Informatics at the University of California, San Francisco [61].

**Figure 6:**
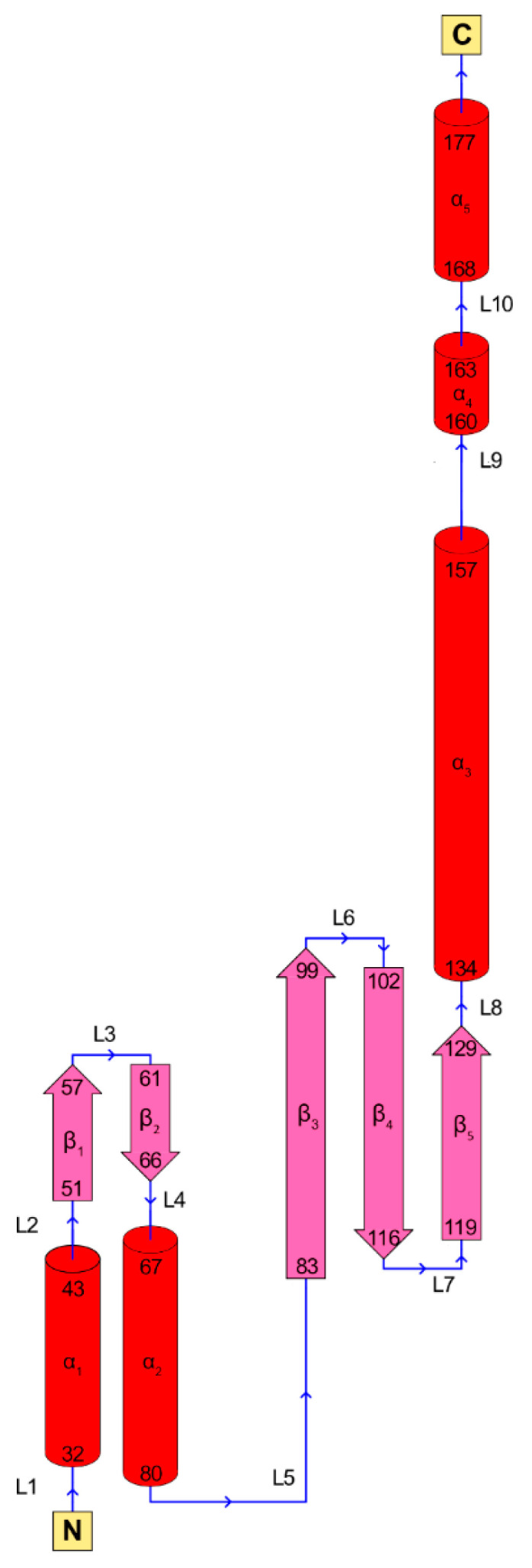
Schematic representation of RAD52 (1–212) monomer folding. Rods and arrows indicate helices and strands, respectively.

RAD52 FL rings have a tendency to form even higher molecular weight (MW) superstructures that interact with other protein functional units in a stacked- or side-by-side fashion. The tendency to form such high-MW superstructures is increased in the presence of DNA [49,51,56,57]. X-ray crystal structures, available for the N-terminal portion of RAD52 (i.e., AA 1–212 or 1–209) (PDB 1H2I, 1KN0, 5JRB, 5XRZ, and 5XS0), have allowed the protein’s DNA-binding and multimerization domains to be characterized [49,58,59,60]. The crystal structure of RAD52’s N-terminal domain shows a ring-shaped undecamer, resembling a mushroom, with a “stem” and a “domed cap” (Figure 5). The stem of each monomer has a β- β- β-α structure, in which the upper parts of the β-strands of all the monomers align side by side, forming the inner part of the channel. The domed cap comprises amino acids flanking the β- β- β-α structure at both the C- and N-terminal portions. The α-helices 1 and 5 (Figure 5; Figure 6) have hydrophobic interactions with the upper part of the β-barrel-like stem, leading to the protrusion of a hairpin loop comprising β1-L3- β2 fragments (the “lobe”). In contrast, the C-terminal portion comprises L9- α4-L10- α5 and is bound by hydrophilic interaction to the flanking monomer of RAD52’s ring.

**Figure 7:**
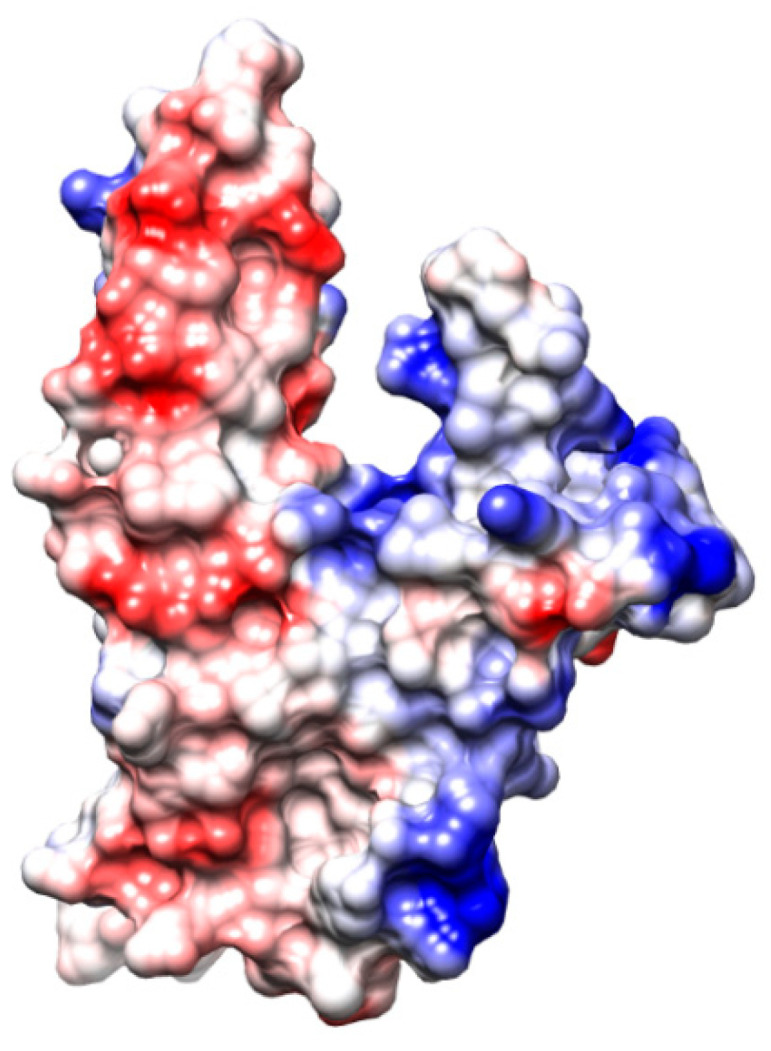
Schematic representation of RAD52 (1–212) monomer surface electrostatic potentials (PDB 1KN0). The representation was carried out using the Coulombic tool of UCSF Chimera, where −10 was set as the minimum (red) and 10 was set as the maximum (blue).

This structure leads to the formation of a negatively charged surface at the top of the flat, domed cap and a positive charge at the bottom of the ring, between the stem and the hairpin loop, representing the protein’s first DNA-binding site (Figure 7). The DNA wraps alongside the outer part of RAD52’s undecameric ring, fitting into this positive cleft [49,62].

In 2008, Kagawa and colleagues identified a second DNA/RNA binding region located close to the entrance of the positively charged surface [63]. This new DNA-binding site has shed more light on RAD52’s potential mechanisms of action, i.e., mediating and promoting ssDNA annealing, homology search, and D-loop formation [49,58,62,63].

Given the significant difference in sequence length, it is surprising that both the N-terminal form of RAD52 and RAD52 FL form ring-shaped oligomers with similar diameters (around 10 nm) [51,52,53,64]. The first ring-model structure of the full-length RAD52 was built by Kagawa and colleagues [49], merging information from RAD52’s N-terminal structure with rough information obtained from dynamic light scattering (DLS), scanning transmission electron microscopy (STEM), and analytical ultracentrifugation (AUC) data on the full-length protein [51,52]. The heptameric ring model of RAD52 FL was built on indirect experimental evidence and two main speculations. First, if RAD52 (1–212) monomers fit in a heptameric ring, the distance between β- β- β-α folds would increase by 1 nm. Second, it was assumed that if the RAD52 FL monomer-monomer interfaces comprised β-barrel structures, then the difference in distance between neighboring monomers could fit two β-sheets better than the RAD52 (1–212) oligomeric structure. Notably, several structure-based prediction techniques allowed researchers to identify possible β-sheet structures in the limited disordered region of the N-terminal portion of RAD52 (1–212) (Val23 to Phe26), Gln221 to Val343, and downstream of the residue Ser346 [49]. As already mentioned above, work has recently been published reporting the 3.5 Å cryo-EM structure of full-length RAD52, suggesting that the oligomerization state of full-length RAD52 is undecameric, as for the N-terminal truncated form of the protein, with, however, a disordered C-terminal domain [55].

Regarding RAD52’s physiological forms, in 1999, Kito and colleagues demonstrated the existence of different RAD52 shorter isoforms with the same DNA binding and homologous pairing activities as RAD52 FL [57]. These similarities were because the RAD52 protein isoforms (AA 1–177 of the human RAD52) share 70% homology at the N-terminal [49] (Figure 4). In agreement with these results, Kagawa and colleagues suggested that the 11-mer ring of the truncated N-terminal RAD52 could be one of the oligomerization states displayed by RAD52 homologs and shorter isoforms [57].

Both the RAD52 FL and the RAD52 N-terminal domain have elevated thermal stability [56]. This feature is probably linked to their oligomeric state and to their propensity to form higher MW ring complexes. Notably, even though the RAD52 N-terminal domain and RAD52 FL have a similar ring structure, the propensity to form high-MW superstructures is greater in RAD52 FL than in the RAD52 N-terminal domain [51,53,56,65]. This may be because the C-terminal domain portion favors intermolecular bonds and hydrophilic interactions between the different protein functional units [51].

### 3.1. DNA/RNA Binding

RAD52 exerts its biological function through DNA and RNA binding, prompting homology search and annealing of DNA strands, and supporting control of genomic stability. The RAD52 mechanism of DNA/RNA binding is not yet elucidated. However, researchers have investigated some features of these binding mechanisms and proposed several models [59,66,67].

The first evidence of DNA binding was reported for yeast Rad52 [68,69]. Several studies, including structural EM investigations, then reported the characterization of DNA-RAD52 binding in human RAD52 [52,53,54,62].

As noted above, protein–DNA interaction is mediated by RAD52’s N-terminal domain, which is therefore critical to RAD52’s DNA-related activity [51,59,70,71] and its intracellular mechanism of action.

**Figure 8:**
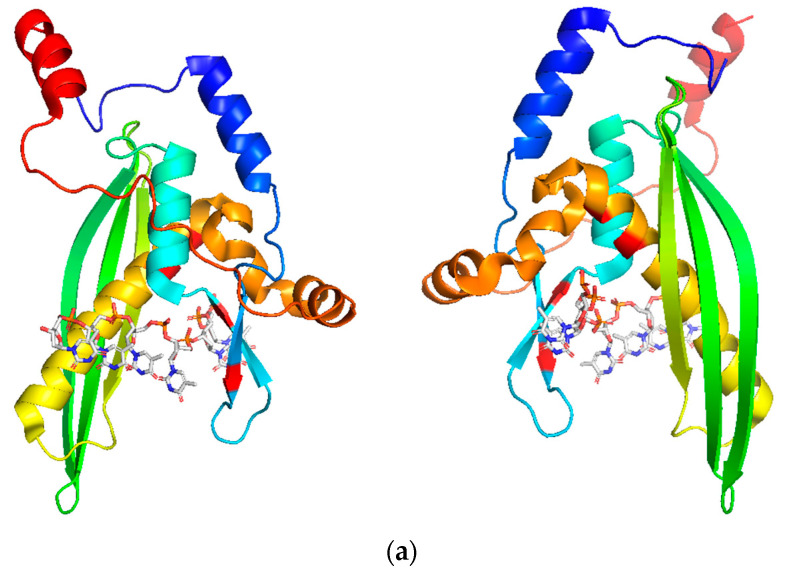

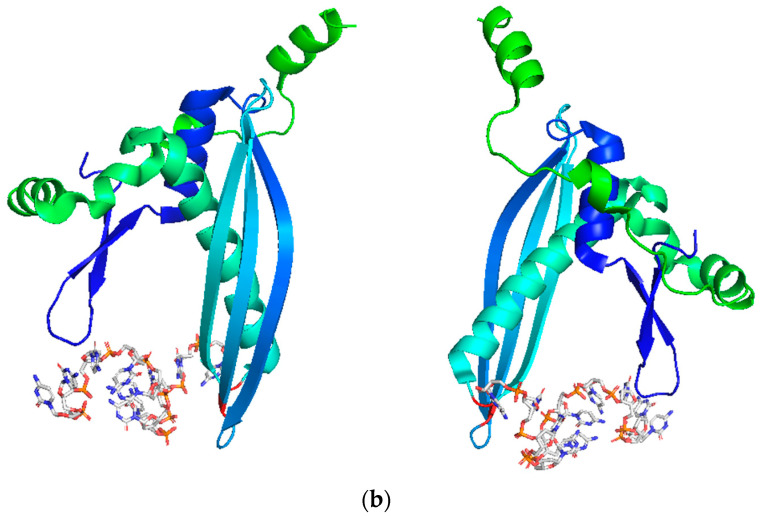
(**a**) Two views of the RAD52 (1–212) monomer in complex with an ssDNA molecule inside its inner binding cleft (PDB 5XRZ); (**b**) two views of the RAD52 (1–212) monomer in complex with an ssDNA molecule inside its outer binding cleft (PDB 5XS0). Structures were prepared using UCSF Chimera software.

Since 2008, two DNA-binding grooves on RAD52’s N-terminal structure have been identified and associated with protein activity [63]. However, the key progress in characterizing the DNA–RAD52 interaction came in 2018, when Saotome and colleagues crystallized the RAD52 N-terminal domain in the presence of ssDNA [59] (Figure 8). The solved crystal structures supported the previously proposed existence of two DNA-binding sites in RAD52 [62,63,72]. Regarding the inner binding site, the single-strand DNA wraps around RAD52, fitting inside a positively charged groove. Each protein monomer should accommodate four nucleotides, with the bases of the base-pairing edges exposed to the solvent, most likely facilitating homology search and annealing to a second single-strand DNA. Furthermore, DNA binding does not appear to affect the protein conformation and oligomerization state, meaning that the inner DNA-binding groove of the RAD52 ring is in a ready state for DNA binding. The DNA inside the groove is stabilized by stacked hydrophilic interactions between DNA bases and Arg55 and Val63 and by electrostatic interactions between the DNA stretched phosphate backbone and the basic amino acids in the DNA-binding site.

Intriguingly, a similar binding mode has been reported for bacterial RecA recombinase, although this protein and RAD52 have different oligomerization patterns and no sequence homology [73]. Hence, this binding mode, as common to these rather diverse ssDNA-binding proteins, could speculatively be generalized to be a common feature of the proteins, which support DNA annealing and base pairing [59].

In the second outer DNA-binding site, reported in the RAD52 N-terminal’s crystal structure (PDB 5XS0) [59], the DNA is buried between two different RAD52 ring structures as a compact, right-handed helix. This suggests the outer DNA-binding site promotes multiple RAD52 ring localizations on a DNA strand, mediating the annealing of DNA strands [63]. Relative to the inner binding site, the outer DNA-binding site has a greater affinity for DNA binding (K_d_ (outer) = 200 nM; K_d_ (inner) = 24 μM). Nevertheless, the two binding sites work cooperatively for DNA binding, with the simultaneous binding of the DNA in the two sites reducing the binding affinity (K_d_) for both sites (K_d_ (outer) ~6 nM; K_d_ (inner) ~100 nM).

Based on these data, Saotome and colleagues suggested that RAD52 may facilitate the annealing and homology search of DNA strands [59]. In particular, in order to anneal, DNA strands must first bind to the outer DNA-binding site of each RAD52 ring before sliding to the inner DNA-binding site. Once DNA strands are in this position, RAD52 rings can move closer, associate with one another, and facilitate the ssDNA annealing and homology search of the two “trapped” DNA strands. This DNA annealing mechanism has also been observed in other single-strand DNA-binding proteins in lower-complexity organisms, such as different types of bacteria [74].

Rothenberg and colleagues previously proposed a similar mechanism of action [67], in which DNA strand annealing occurs via the interaction of two or more RAD52 rings, accommodating ssDNA with the bases presented outward. The association of complexes can then facilitate the pairing of bases and stabilization. If complementarity is present, annealing begins with 3–4 bases of nucleation length, and the two or more nucleoprotein complexes can roll around each other with an energy-favorable duplex formation-driven force [60,67].

**Figure 9:**
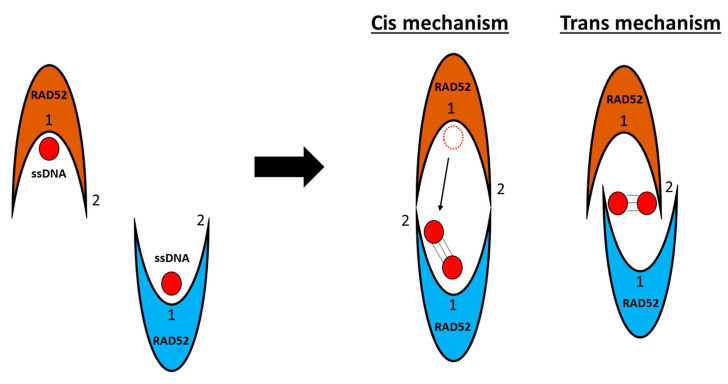
Schematic representation of the two postulated cis and trans mechanisms of RAD52-mediated homology search. The dashed line circle represents the original position of one of the two DNA strands in the deep inner binding groove (1) of one of the two nucleoproteins that is then temporarily placed in the second binding site (2) of the second nucleoprotein upon homology search (solid line circle).

Notably, in 2010, Grimme and colleagues studied DNA-RAD52 interaction mechanisms and postulated two possible mechanisms for homology search, both cis and trans (Figure 9). In the cis mechanism, a portion of one DNA strand can emerge from the deep inner binding groove and be temporarily placed in the second DNA-binding site of the second interacting nucleoprotein. In the trans mechanism, both DNA strands can be pulled out from the inner binding site and moved up to the secondary binding sites of their respective rings to start the homology search and annealing [72]. Notably, the most effective annealing of DNA strands occurs between two RAD52-ssDNA nucleoprotein complexes and not between RAD52-ssDNA and protein-free DNA [72].

In the same work, Grimme and colleagues also suggested that RAD52 takes part in DNA recombination activity, facilitating RPA protein release from the DNA filament [72]. Specifically, it was speculated that, thanks to its ability to form complexes with RPA, RAD52 can remove RPA from DNA and facilitate RAD51 loading on the DSB site, as already reported for yeast Rad52 [72]. This study suggested that the C-terminal domain of RAD52 may regulate the RAD52-RAD51-RPA interaction on DSB sites and thus may play a critical role in DNA strand annealing. In 2017, Ma and colleagues also confirmed RAD52’s role in RPA turnover on DSB sites [75]. However, the mediator activity of RAD52 for RAD51 loading on DNA and interaction with RPA in humans needs to be further supported by other in cellulo and in vivo evidence to be definitely validated.

As reported by Kagawa and colleagues in 2001 and 2008, RAD52 binds dsDNA, promoting D-loop formation. This was later corroborated by other studies, highlighting the importance of both RAD52 DNA-binding sites for protein activity in homology search and strand invasion [5,62,63,76].

Interestingly, recent studies report that RAD52 can bind not only DNA but also RNA [36,39]. ssRNA and ssDNA show the greatest binding affinity for RAD52, with a significantly lower affinity observed for the double-stranded substrates [5,36]. Among the double-stranded substrates, RNA-DNA hybrids have a more efficient binding to RAD52 relative to dsRNA and dsDNA. Finally, RAD52 has a greater affinity for R-loop structures than hybrid structures. These data are in line with RAD52’s key role in specific DNA repair mechanisms, namely RNA-template recombination repair, as described above. Notably, while it is clearly reported that RAD52 shows the highest affinity for ssDNA, for the other substrate, no systematic quantitative studies have been published yet. Thus, the lack of robust data must be taken into consideration for assertions regarding DNA and RNA binding.

### 3.2. Post-Translational Modification

Recent years have seen many studies and hypotheses about the post-translational modifications required for RAD52 to perform its function [2].

RAD52 acetylation is a critical modification that regulates RAD52’s function [77,78]. Specifically, non-acetylated RAD52 can accumulate at DSB sites, where it is recruited, but it dissociates prematurely. In the absence of RAD52 acetylation, RAD51 also dissociates prematurely from DSB sites, impairing HR [77]. Moreover, SIRT1-SIRT2 deacetylase depletion induces effects equivalent to RAD52 depletion but without affecting SSA and NHEJ repair [78]. The recruitment of RAD51 to DSB sites is also affected by SIRT2 or SIRT3 depletion but not by RAD52 deacetylation [78]. These preliminary studies suggest that acetylation and deacetylation of RAD52 may be a regulatory mechanism controlling protein–protein interactions between RAD52 and HR-related proteins in multiple HR steps. Nevertheless, further investigations are necessary to clarify these mechanisms.

Based on sequence homology with yeast Rad52, researchers have suggested that the human RAD52 protein undergoes sumoylation modification, which should not affect protein–protein interactions and may only affect DNA binding and strand annealing [2,79]. Nevertheless, RAD52’s sumoylation site was identified in its nuclear localization signal (NLS) region at the C-terminal, suggesting that sumoylation could play an important role in RAD52 nuclear transport [80].

Finally, phosphorylation of RAD52 at Tyr104 enhances ssDNA annealing activity while lowering RAD52’s dsDNA-binding ability. Additional studies on constitutively active oncogenic BCR-ABL1 kinase have demonstrated that RAD52 phosphorylation facilitates its nuclear localization and stimulates SSA repair in leukemia cells [81,82,83]. However, phosphorylation of Tyr104 is not strictly required for RAD52 to exert its DNA-binding activity [84].

## 4. RAD52 in Cancer

Different studies have shown that there is a clear correlation between RAD52 misregulation and cancer. Overall, they reported different expression states of RAD52 depending on the cancer type but showed contradictory expression patterns. The correlation between specific RAD52 genetic variants and cancer development has also been established in several studies.

In particular, a direct correlation between specific RAD52 single-nucleotide polymorphisms (SNP) and tumorigenic risk was speculated, for instance in hepatitis B virus (HBV)—hepatocellular carcinoma (HCC) [85] and in colorectal cancer [86]. Additionally, a recent work reported, for example, that the S346X mutation in RAD52 was found to correlate with a significant reduction in breast and ovarian cancer risk for germline BRCA2 mutation carriers, supporting the idea that RAD52 defects in BRCA-mutated carriers could lead to a lower risk of tumor development [87].

It is interesting that depending on the different types of cancer, RAD52 was found to be up- or down-regulated, and RAD52 levels might correlate with a good or poor prognosis for the patients. For instance, cervical and rectal cancer cells with low RAD52 expression were associated with poor response to platinum-based chemotherapies and increased resistance [88,89]. Moreover, low expressions of RAD52 were shown to correlate with poor overall survival for urothelial cancer patients [89,90].

On the other end, RAD52 overexpression was reported in many other cancer types, sustaining the speculation that RAD52 is important to enhance the viability of cancer cells and the dysregulation of cancer cells’ DNA repair mechanisms [91]. RAD52 overexpression was reported to correlate with hepatocarcinogenesis in TGF-α/c-myc mice [92], and its depletion or inhibition was reported to decrease cancer incidence and exert antileukemic effects in ATM-deficient mice [93] and in acute myelogenous leukemia (AML), B-cell acute lymphoblastic leukemia (B-ALL), and T-cell acute lymphoblastic leukemia xenografts with low BRCA1/2 expression [84].

In another study, RECQL4-deficient breast, colon, and lung cancer cells that presented significant RAD52 upregulation displayed more sensitivity to ionic radiation [94].

Additionally, high expression of RAD52 correlates with a poor life span for rectal cancer patients [89,90].

Finally, expression studies performed on RAD52 genetic variants in lung squamous cell carcinoma (LSCC) have demonstrated that variations in RAD52 protein expression correlate with the risk of non-small cell lung cancer (NSCLC) and that RAD52 depletion increases the death of cells undergoing carcinogenic transformations and in vivo antitumoral activity [95]. These pieces of evidence further support the idea that RAD52 inhibition is a promising approach to targeting specific types of cancer (depending on RAD52 expression level) and pursuing precision and personalized medicine.

## 5. RAD52 Inhibition in Synthetic Lethality Therapies

Due to their intrinsic genome mutations, cancer cells frequently show inactivation of many of the canonical DNA repair pathways. However, even if they accumulate high levels of spontaneous and drug-induced DNA damage, they survive by relying on alternative DNA repair pathways [96]. As discussed above, RAD52 mediates many of these alternative pathways [1,5], and while not normally essential for cell survival, RAD52 is essential in cancer conditions where other DSB repair-related proteins are mutated [9,10]. This makes RAD52 an attractive target for synthetic-lethality-based selective anticancer therapy.

Indeed, RAD52 has an important oncogenic role in mediating many DDR pathways on which cancer cells rely when canonical pathways are disrupted [97,98]. Inhibiting RAD52 induces synthetic lethality in many cancer cells with defective DNA repair-related proteins, such as BRCA1, BRCA2, PALB2, XAB3, and RAD51 paralogues (i.e., RAD51B, RAD51C, RAD51D, XRCC2, XRCC3) [9,10,99,100]. Of these DSB repair mediator proteins, the synthetic lethality relationship between RAD52 and BRCA1/2 has been thoroughly investigated in cells in recent years [101].

Researchers have devoted considerable effort to identifying and characterizing RAD52 inhibitors for synthetic lethality strategies in BRCA1/2-depleted cancers and in cells with drug-induced BRCA1/2 depletion. A strategy that, for instance, showed some promising results in the past but that was not pursued forward consisted in the use of microRNA (in particular miR-302a and miR-210) to regulate RAD52 protein expression at the post-transcriptional level by specifically binding to the 3′-untranslated region (3′-UTR) of its mRNA [102,103].

Most campaigns to discover RAD52 inhibitors use virtual screening, docking the molecules in “druggable” RAD52 pockets that are considered important for their activity. Additionally, high-throughput screening (HTS) assays on RAD52 based on biochemical or biophysical assays (such as fluorescence polarization (FP) [65] and electrophoretic mobility shift assay (EMSA) [65,84,104,105]) are also widely used. Most of these assays measure compound binding by using a fluorescently labeled probe (e.g., cyanine5-labeled 30 nucleotides of ssDNA) to detect the inhibition of the DNA-RAD52 interaction via changes in electrophoretic mobility or donor-acceptor energy transfer. Isothermal titration calorimetry (ITC) [65], microscale thermophoresis (MST) [105], surface plasmon resonance (SPR) [106], RAD52-pull down assays [107], and WaterLOGSY (NMR technique) [108] have also been used to validate RAD52 inhibitors via direct binding. In vitro assays are then frequently used to assess the inhibition of RAD52, SSA, or HR activity in the presence of inhibitors. Further characterizations of the most promising compounds often involve cellular experiments focused on RAD52 molecular activity, especially in BRCA-deficient cancer cells. Therefore, cell viability and RAD52 foci formation in BRCA1/2-deficient cancer cells are often performed to indirectly demonstrate target engagement and synthetic lethality [9,10]. Finally, cell-based DSB repair assays (SSA or HR) have been used to investigate findings from in vitro screenings in cells [65,104,106,109].

**Figure 10:**
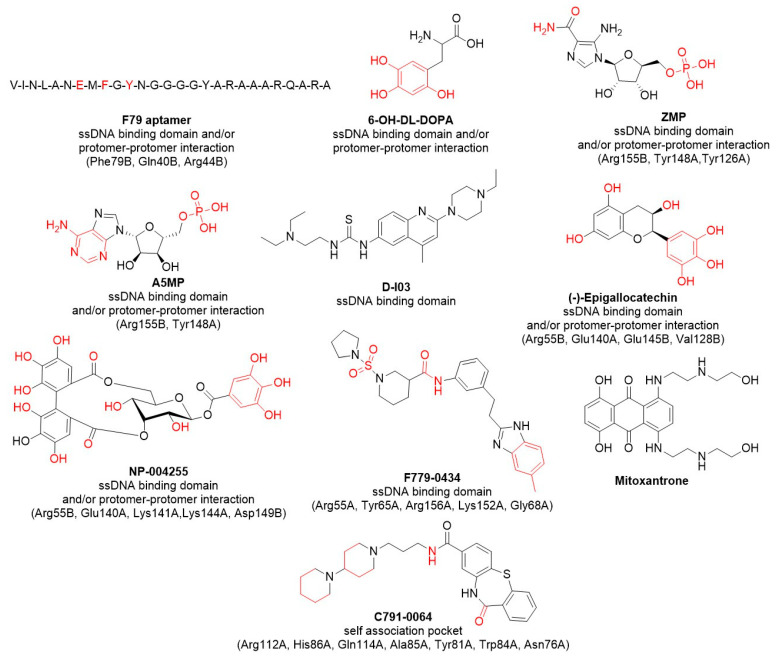
Structures of most recent and relevant inhibitors targeting RAD52. Inhibitors names are in bold, with their binding pockets with the amino acids of interest in brackets; A or B following the amino acid numbers indicates if the amino acid belongs to RAD52 protomer A or B; functional groups that establish specific interactions with amino acids are reported in red; Phe-Phenylalanine; Gln-Glutamine; Arg-Arginine; Tyr-Tyrosine; Glu-Glutamate; Val-Valine; Asp-Aspartate; Lys-Lysine; Gly-Glycine; Asn-Asparagine; Trp-Tryptophan; His-Histidine; Ala-Alanine.

There are no FDA-approved RAD52 inhibitors on the market yet, and no RAD52 inhibitors are reported to be in clinical development yet (ClinicalTrials.gov). However, the inhibitors reported to date suggest that the most druggable regions of the protein are the ssDNA-binding domain and the oligomerization interface, which are interconnected. The collected data suggest a few general guidelines for the rational design of new RAD52 inhibitors. Since these grooves and interfaces are characterized by basic amino acids (Lys and Arg), almost all the reported inhibitors have acid portions (trihydroxylated phenolics, sulfonamides, phosphates, and amides). However, hydrophobic interactions also appear to be important, and aliphatic and/or aromatic portions are often used. Below, we describe the most recent and relevant RAD52 inhibitors and discuss possible structure-activity relationships (SAR), given RAD52’s biochemical and cellular activities (summary in Figure 10 and Table 1).

### 5.1. F79 Peptide Aptamer

In 2013, Skorski and colleagues developed F79, a peptide aptamer and the first RAD52 inhibitor ever described [84]. F79 was designed to inhibit RAD52 and exert synthetic lethality in BRCA-disrupted and/or HR-mutated tumor cells [84]. The peptide aptamer was designed after mutagenic assays reported the fundamental role of the residue Phe79 in RAD52-DNA binding and in RAD52 protomer-protomer hydrophobic interactions. Using computational methods, the Skorski group designed a peptide aptamer containing the 13-amino-acid sequence surrounding Phe79 (F79) in RAD52’s α2 helix. They reported a significant abrogation of RAD52-DNA-binding activity after treatment with F79.

In particular, two residues were fundamental to the aptamer’s binding ability: Glu77, which established a salt bridge with Arg44; and Tyr81, which established an H-bond interaction with Gln40 [84].

Cell studies have reported that F79 selectively killed BRCA-deficient leukemia cells with a low risk for normal cells (EC_50_ < 5 μM). Indeed, synthetic lethality was observed in CML cells with the BCR-ABL1 mutation (BRCA1 downregulated), acute promyelocytic leukemia (APL) cells with the PML-RAR mutation (RAD51C downregulated), and other patient-derived leukemia cells with epigenetic modifications resembling the BRCA-deficient phenotype [84]. In in vivo tests, F79 treatment significantly extended the life spans of severe combined immunodeficiency (SCID) mice with BCR-ABL1-positive leukemia. Additionally, F79 treatment induced synthetic lethality in BRCA1/2-mutated breast, pancreatic, and ovarian cancer cells and exerted synergistic effects with imatinib (approved for BCR-ABL1-positive leukemia) and ATRA (for PML-RAR-positive leukemia) [84].

In 2019, F79 was observed to inhibit the proliferation of acute myeloid leukemia (AML) cells and to promote cell apoptosis in AML cells treated with etoposide. Moreover, the RAD52 aptamer also affected the expression and activation of the apoptotic signal protein STAT3 [110]. F79 was therefore proposed as a novel therapy against STAT3 continuous activation in myeloid leukemias [111,112]. However, there have been no further reports on subsequent development or delivery studies with this peptide.

### 5.2. 6-OH-DOPA

In 2015, the Chandramouly group discovered 6-OH-DOPA, the first small molecule RAD52 inhibitor [65]. Chandramouly and colleagues set up an HTS in a library of drug-like compounds (Sigma Lopac), using the FP method to detect molecules that could affect the DNA-RAD52 interaction. 6-OH-DOPA was identified and further characterized with other biophysical studies. 6-OH-DOPA emerged as a disruptor of both the DNA-RAD52 interaction (IC_50_ of 1.1 μM) and of the RAD52 heptamer and undecamer structures. 6-OH-DOPA inhibited SSA with no or little effect on other mechanisms such as HR and D-NHEJ in BRCA-proficient cells [113]. Moreover, 6-OH-DOPA selectively inhibited cell proliferation in BRCA1-depleted triple-negative breast cancer (TNBC) cells and in BRCA-deficient AML and CML patient cells.

As a catechol, 6-OH-DOPA is potentially a pan-assay interference compound (PAINS, i.e., classes of compounds that have been recognized to react nonspecifically against many different substrates and may give false positive readouts in HTS assays) [114]. However, the in vitro and cell-based data showed that its activity against RAD52 is somewhat specific. Despite these promising results, 6-OH-DOPA is unsuitable for anticancer therapy because it is a dopaminergic toxin that contributes to Parkinson’s disease and the degeneration of mitral neurons [115]. However, SAR studies could shed light on modifications to remove its dopaminergic toxin activity. Although a few other catechols were tested, no other significant synthetically accessible modifications were investigated. For example, 6-OH-DOPA is a racemic mixture, and the separation of its two enantiomers could indicate the beneficial activity of one enantiomer over the other.

### 5.3. A5MP/ZMP

In 2016, Sullivan and colleagues took an alternative approach to discovering RAD52 inhibitors to achieve synthetic lethality [104]. They performed molecular docking studies and virtual screenings of a drug-like compound library (http://zinc.docking.org/catalogs/ncip; containing 139, 735 structures) and 1217 FDA-approved drugs (http://zinc.docking.org/catalogs/fda) in order to identify compounds able to inhibit the RAD52-ssDNA interaction. The most promising compounds were adenosine 5′-monophosphate (A5MP) and 5-aminoimidazole-4-carboxamide-ribonucleotide (AICAR) 5′ monophosphate (ZMP), which inhibited ssDNA binding to RAD52 and the growth of BRCA1-deficient HCC breast cancer cells. AICAR (a ZMP precursor [116]) was also effective in Capan-1 pancreatic cancer cells but inactive in RAD52-deficient cells. As expected, BRCA1/2 reconstitution completely abrogated the sensitivity to the compounds. Both AICAR and AMP inhibited RAD52 nuclear foci formation in BRCA-1-deficient leukemia cells after cisplatin-induced DNA damage.

Finally, docking study refinements suggested that both A5MP and ZMP localize at the intersection between two monomers of RAD52, interacting with its DNA-binding domain [104]. Interestingly, the two molecules bind in a similar manner, with the aminopyrimidine moiety of A5MP and the amide group of ZMP both interacting with Tyr126. In addition, the phosphate groups of both compounds assume a similar orientation and form an H-bond interaction with Thr148. Furthermore, Arg55 and Lys141 were potential target residues for additional H-bond interactions. This could be achieved with several structural modifications on ZMP and A5MP. A phosphate bioisostere approach might be a viable strategy for designing more permeable and ultimately more potent RAD52 inhibitors [117].

### 5.4. D-I03

In 2016, researchers used an HTS with a fluorescence-quenching assay to identify a series of novel RAD52 inhibitors [106]. The assay tested how well the compounds inhibited RAD52 activity on complementary strand annealing and invasion of ssDNA into a homologous duplex of DNA. D-I03 is a promising RAD52 inhibitor, showing the highest inhibitory effect on D-loop formation in vitro on several BRCA1/2-deficient cell lines (Capan-1, MDA-MB-436, and UWB1.289) and on BRCA1-deficient BCR-ABL-positive CML cells from patients. No D-I03 effect was observed in BRCA-proficient cells. Additionally, D-I03 inhibited RAD52-mediated SSA foci formation after cisplatin treatment. Finally, D-I03’s binding affinity K_d_ for RAD52 was 26 μM, as determined by SPR, supporting the idea that the effects observed in cells and in vivo could correlate with D-I03 direct binding to RAD52 and may interfere with its DNA-binding activity [106].

The Skorski group later demonstrated the efficacy of D-I03 also in vivo: they reported that D-I03 was effective in reducing the growth of BRCA1-deficient xenograft tumors in mice, and its effect was even more pronounced when in combination with PARP1 inhibitor Talazoparib [96].

Although D-I03 was the most potent compound identified in the HTS and its effect was also reported in vivo, its thiourea scaffold posed drawbacks such as poor solubility and metabolic and chemical instability [118]. The HTS also identified other compounds belonging to a structurally diverse scaffold (3-[(2-(metoxyanilino)aminoquinazolin-4-yl)amino]propan-1-ol) with promising in vitro and in cellulo activity, creating more opportunities to explore this chemical space [106].

### 5.5. Epigallocatechin; Epigallocatechin-3-Monogallate; NP-004255

In 2016, Hengel and colleagues conducted a fluorescence resonance energy transfer (FRET)-based HTS of the MicroSource SPECTRUM collection (MicroSource Discovery systems) associated with a virtual screening campaign. They identified two RAD52 inhibitors (Epigallocatechin; Epigallocatechin-3-monogallate) predicted to bind to the DNA-binding groove running around the RAD52 oligomer [108]. These compounds also inhibited RAD52’s binding to RPA-coated ssDNA and its ability to anneal ssDNA. NMR waterLOGSY analysis confirmed their binding to RAD52 [119]. Epigallocatechin also inhibited RAD52/MUS81/EME1-dependent DSB formation in hydroxyurea-treated cells and in checkpoint-deficient cells. This suggests that RAD52’s ssDNA-binding activity is necessary to recover stalled replication forks [26,108]. Finally, epigallocatechin significantly reduced the viability of BRCA2-depleted or MUS81-depleted cells under conditions of replication stress [108]. To corroborate their hypothesis about the inhibitors’ mechanisms of action, Hengel and colleagues also proposed and validated a novel in silico screening campaign based on their HTS results. This led them to discover NP-004255 (corilagin) as an inhibitor of the RAD52-ssDNA interaction and to confirm corilagin’s properties with biophysical assays [108]. Corilagin is a macrocyclic ester comprising three trihydroxylated phenolic moieties. These aromatic rings are also found in epigallocatechin and epigallocatechin-3-monogallate, with which corilagin shares the binding pocket. The phenolic rings exploit an interstitial water network that, together with pivotal interactions with Lys141 and Lys144, Arg55, Glu140, and Glu145, assures optimal binding to the ssDNA-binding groove.

### 5.6. F779-0434

In 2018, Li and colleagues proposed another virtual screening campaign [107]. They defined the amino acids required for ssDNA-binding by RAD52 and performed a docking campaign of 47,737 compounds. F779-0434, predicted to bind in RAD52’s ssDNA-binding groove, was one of the best candidates for further development. Similarly, to the previously reported inhibitors, lysine (Lys152) acted as a key amino acid residue and played an important role in the F779-0434 binding of RAD52. Indeed, this compound demonstrated a promising disruption of the RAD52-ssDNA interaction in vitro and inhibited the growth of BRCA2-deficient cells (Capan-1) with an insignificant effect on BRCA2-proficient cells (BxPC3 cell line) [107].

### 5.7. Curcumin

Tseng and colleagues recently used cell-based and in vivo assays to study curcumin as a novel inhibitor of RAD52 in BRCA-2-deficient cell lines [120]. Motivated by their previous evidence in budding yeast Rad52 [121], they confirmed that curcumin inhibited RAD52 expression in human cancer cells too, using the MCF-7 cell line treated with the irinotecan (CPT-11) damaging agent and then with different curcumin conditions. Curcumin impaired CPT-11-induced RAD52 upregulation (both transcriptionally and post-translationally) and RAD52 foci formation. Curcumin inhibited growth in BRCA-deficient cell lines (MCF-7, siBRCA2, and Capan-1) at a higher rate than in their normal counterparts. Curcumin also sensitized BRCA-deficient cells to CPT-11, with no significant effect on their normal counterparts. Cellular studies with siBRCA2 MCF-7 cells also demonstrated that curcumin impairs HR in BRCA-deficient cells. Finally, in in vivo experiments, curcumin sensitized BRCA2-knockout MCF-7 cells to CPT-11 chemotherapy in tumor xenografts.

Interestingly, Tseng and colleagues proposed curcumin’s mechanism of action after conducting a docking study that showed curcumin can make several hydrogen bonds with Phe26 of different RAD52 monomers, leading to a conformational change at the N-terminal [120]. However, curcumin was recently recognized as a pan-assay interference compound (PAINS), so these promising results must be interpreted in light of a potential nonspecific effect [114,122].

### 5.8. C791-0064

Yang and colleagues conducted a virtual screening campaign with a previously used computational algorithm but selected a novel binding pocket (i.e., self-association domain, critical for forming the functional ring unit) [105]. C791-0064 was the most promising novel RAD52 inhibitor of the 66,608 compounds screened. The docking studies suggested that C791-0064 docks in RAD52’s self-associating binding site, making hydrogen bonds with Arg112, His86, and Ser67 inside the groove and Tyr81 and Asn76 outside the groove. C791-0064 was validated as a novel RAD52 inhibitor in cellular and in vitro studies. Compared to their normal counterparts (BxPC3), BRCA2-deficient cell lines (Capan-1 or shBRCA2-BxPC3) were more sensitive to C791-0064 treatment in clonogenic and cell viability assays. Moreover, C791-0064 does not reduce the viability of siRAD52/sh-BRCA2-BxPC3 cell lines compared with untreated siRNA/sh-BRCA2-BxPC3 controls, demonstrating C791-0064′s selectivity for RAD52. Additionally, more apoptosis and DNA damage were observed in BRCA2-deficient cells than in wild-type cells. BRCA2-deficient cells treated with C791-0064 had a significant increase in the DNA damage biomarker γH2AX and in apoptosis markers, suggesting an inefficient DNA reparation mechanism after C791-0064 treatment in this BRCA-deficient condition. In vitro assays demonstrated that C791-0064 disrupted the oligomeric form of RAD52 and inhibited its ssDNA annealing activity [105].

### 5.9. Quinacrine, Mitoxantrone, Doxorubicine

In a different approach to discovering RAD52 inhibitors, a recent study targeted the RPA-RAD52 interaction rather than RAD52 oligomerization or DNA-binding sites [107]. Using FluoIA-based HTS, the researchers identified quinacrine, mitoxantrone, and doxorubicine as the most potent compounds, with an EC_50_ of 97.7 μM, 29.7 μM, and 10.1 μM, respectively. Using co-immunoprecipitation of RAD52 and RPA, mitoxantrone disrupted the RPA-RAD52 interaction. All three compounds inhibited the cell viability of different HR-disrupted ovarian and breast cancer cell lines, including some PARPi-resistant cell lines, at a greater rate than their HR-proficient counterparts. However, quinacrine was less selective toward HR-deficient ovarian cancer cells. Using a U20S SSA cell-based assay [113], mitoxantrone selectively inhibited RAD52-dependent SSA better than HR at low doses and was comparable at higher concentrations. Furthermore, in BRCA2-deficient cells after X-ray radiation, mitoxantrone inhibited RAD52 foci formation without affecting radiation-induced RAD51 foci formation. These results indicate that RPA-RAD52 interaction could be a promising therapeutic target for HR-disrupted cell lines [109] and that mitoxantrone is a potential novel RAD52 inhibitor for new synthetic lethality strategies. Mitoxantrone is a chemotherapeutic drug that uses its flat, polycyclic aromatic rings to intercalate DNA and so induce DSBs. Moreover, it specifically targets the topoisomerase IIα isoform. Therefore, further biophysical studies are needed to confirm and better characterize its binding to RAD52 and consequently attribute the enhanced cellular toxicity to this activity.

## 6. Conclusions and Future Perspectives

RAD52 has emerged as a novel druggable target for innovative therapies based on synthetic lethality (e.g., in BRCA2-mutated cancers). Although further studies will be required to achieve a comprehensive description of its structure and mechanism, cutting-edge pieces of evidence suggest its potential for precision medicine. Specifically, RAD52 seems inessential for the viability of normal cells. However, in the presence of specific mutations related to HR DNA repair, RAD52 inhibition affects tumor cell viability and induces synthetic lethality. RAD52 is therefore a validated target for novel synthetic lethality strategies based on both PARPi and RAD52i in order to overcome resistance and increase the effectiveness of anticancer therapies. Only one peptide and a very limited number of small molecule inhibitors have been reported, with most of them targeting the DNA-binding interaction or the self-oligomerization domain. For various reasons related to selectivity and toxicity, none of these inhibitors has yet entered the preclinical or clinical phases. Nevertheless, there are still great opportunities to design new molecules and identify new druggable pockets. First, the RAD52 C-terminal domain 3D structure is not yet available, but the solved structure would certainly give new hints for identifying novel pockets and protein–protein interaction patterns, such as the interactions with RAD51 and RPA. Recently, three inhibitors were reported to disrupt the RPA-RAD52 interaction, but they were identified via biochemical assays [109]. A high-resolution 3D structure of RAD52 FL would also boost and rationalize the targeting of this pocket. In addition to these main protein–protein interactions, RAD52 establishes potentially targetable interactions with other proteins, as reported in several databases (e.g., https://string-db.org/, http://www.interactome-atlas.org/, accessed on 1 February 2023). Moreover, many drug discovery approaches have not yet been explored, including fragment-based screening, aptamers, PROTACS, and a more thorough peptidomimetics screening and development, which would be more suitable for competing with large protein–protein interaction surfaces.

Given the state of the art in RAD52 research activity, we recognize that there is still a long way to go to characterize RAD52’s structure and mechanism of action. Nevertheless, once acquired, this knowledge will foster new drug discovery approaches and effective drug discovery campaigns to exploit this fascinating and challenging target.

## Figures and Tables

**Table 1:** RAD52 inhibitors.

Inhibitor	Initial Screening (HTS)	In Vitro Assays	In Cellulo Assays	In Vivo Assays	Group [Ref]
F79 aptamer	Rational design after mutagenic assay (ssDNA groove and protomer-protomer interaction)	RAD52-ssDNA (EMSA)	BRCA1/2± cell survival (viability and clonogenic: BRCA1/2− cells over BRCA1/2+ and combination with PARPi and leukemia standard treatments); foci (γH2AX: increase at 5 μM); apoptosis (flow cytometry: increase at 5 μM)	PARPi + F79 (2.5 mg/kg/day) synergic against BRCA1-deficient primary AML xenograft in NSG Mice	T. Skorski [84,96]
6-OH-DOPA	RAD52-ssDNA (FP: 18,304 cpds – Sigma Lopac)	RAD52-ssDNA (FP: IC_50_ = 1.6 μM, EMSA); DSB repair (ssDNA annealing); RAD52 binding (ITC: K_d_ = 17.8 μM); oligomer dissociation (DLS; native gel analysis)	BRCA1/2± cell survival (viability and clonogenic: BRCA1/2− selective over BRCA1/2+); DSB repair (SSA selective over HR, NHEJ); foci (RAD52: decrease at 10 μM, γH2AX: increase at 10 μM); apoptosis (flow cytometry: increase at 20–40 μM); siRNA-RAD52 (western blot)	none	R. T. Pomerantz [65]
ZMP/A5MP	Virtual screening (docking – PDB: 1KN0 ssDNA groove: 1217 FDA and 139,735 NCI drug-like cpds – ZINC library, DOCK6.6 software)	RAD52-ssDNA (EMSA)	BRCA1/2± cell survival (growth rate: BRCA1/2− selective over BRCA1/2+); DSB repair (SSA: decrease at 20 μM); foci (RAD52: decrease at 20 μM)	none	T. Skorski [104]
D-I03	DSB repair (ssDNA: 93,672 cpds from Diversity Oriented Synthesis (DOS) library and 279,231 cpds from Molecular Libraries Probe Center Network (MPLCN) - Broad Institute)	DSB repair (ssDNA annealing: IC_50_ = 5 μM, D-loop: IC_50_ = 8 μM); RAD52 binding (SPR: K_d_ = 25.8 μM)	BRCA1/2± cell survival (viability and clonogenic: IC_50_ = 14.5 μM for BRCA1/2− cells over BRCA1/2+ and combination with PARPi); foci (2.5 μM ≈ 2.0-fold reduction of RAD52 and not of RAD51); DSB repair (SSA, 30 μM ≈ 3.4-fold reduction and selective over HR)	PARPi (talazoparib)+D-I03 (50 mg/kg/day) synergic against BRCA1-deficient solid tumor growth in nude mice	A. V. Mazin [96,106]
Epigallocatechines (NP-004255)	RAD52-ssDNA (FRET: 2320 cpds from MicroSource SPECTRUM collection); Virtual screening (docking – PDB: 1KN0 ssDNA groove: AnalytiCon Discovery MEGx Natural Products Screen Library)	RAD52-ssDNA (FRET: IC_50_ = 1.5 μM); DSB repair (ssDNA annealing: IC_50_ = 7 μM); RAD52 binding (WaterLOGSY);	BRCA2± cell survival (viability); DSB repair (comet assay); siRNA-RAD52 (western blot)	none	M. A. Spies; M. Spies [108]
F779-0434	Virtual screening (docking – PDB: 5JRB ssDNA groove: 47,737 cpds Targeted Diversity Library (TDL) – Chemdiv database, DOCK6.5 software)	RAD52-ssDNA (pull-down assay: 50% disruption at 5 μM);	BRCA2± cell survival (viability: BRCA2− selective over BRCA2+, 50% growth inhibition at 10 μM)	none	R. Sun; Q. Zhao [107]
C791-0064	Virtual screening (docking – PDB: 5JRB self-association pocket: 66,608 cpds - Chemdiv database, DOCK6.5 software)	RAD52-ssDNA (EMSA); DSB repair (ssDNA annealing); RAD52 binding (MST);	BRCA2± cell survival (viability and clonogenic: IC_50_ = 29 μM for BRCA2− cells over BRCA+); siRNA-RAD52 and shRNA-BRCA (western blot); foci (γH2AX: increase at 40 μM); apoptosis (flow cytometry: increase at 40 μM, western blot)	none	J. Li [105]
mitoxantrone	RPA:RAD51 PPIs (FluorIA: 335 cpds – SellekChem, 1200 cpds - Prestwick, 100,000 cpds – Chembridge: EC_50_ = 30 μM)	-	BRCA1/2± cell survival (viability: EC50 = 0.1–2.5 μM for BRCA2− cells over BRCA+); DSB repair (SSA reduction and selective over HR up to 6 nM); apoptosis (western blot: increase at 0.2 μM); foci (RAD52: decrease at 3 nM over RAD51); RPA:RAD51 PPIs (co-immunoprecipitation)	none	T. Bessho; G. E. O. Borgstahl [109]

BRCA1/2± cells indicates whether the cells are BRCA-proficient or BRCA-deficient. Cpds—compounds; NSG mice—NOD scid gamma mice; PPIs—protein–protein interaction.
